# Confronting the Unseen: A Journey Through Primary Intra-orbital Squamous Cell Carcinoma

**DOI:** 10.7759/cureus.59598

**Published:** 2024-05-03

**Authors:** Isabella Korchnoy, Juan Gabriel Jimenez, Guillermo Izquierdo-Pretel

**Affiliations:** 1 Internal Medicine, Jackson Memorial Hospital, Miami, USA; 2 Internal Medicine, Florida International University, Herbert Wertheim College of Medicine, Miami, USA

**Keywords:** intra-orbital mass, orbital mass, primary squamous cell carcinoma, primary orbital squamous cell carcinoma, orbital squamous cell carcinoma

## Abstract

Cases of squamous cell carcinoma (SCC) that are primary in origin occur even more infrequently due to the lack of squamous epithelium that is typically present in the orbital region. When SCC occurs in the orbit, it is more commonly due to invasion or metastasis from a local site. We report an uncommon case of intra-orbital SCC in a 74-year-old male, which is likely of primary origin. Brain, face, orbital, and neck magnetic resonance imaging proceeded to gather more information on the extent of the patient’s orbital malignancy, which showed significant orbital burden and intracranial extension. The biopsy was performed with final pathology results showing moderately differentiated SCC. The patient was discharged with a follow-up with oncology for chemotherapy and a follow-up with oculoplastics for surgical intervention in nine months, after completing a course of chemotherapy with irradiation. We provide this case to shed insight into the difficulties associated with the extremely uncommon occurrence of primary SCC of the orbit.

## Introduction

Squamous cell carcinoma (SCC) of the orbit is a very uncommon finding and typically results in secondary to perineural invasion from local skin lesions or metastasis through hematogenous spread from a distant site. Other reports suggest a malignant transformation of a lacrimal gland or orbital cyst [1-4,5]. Primary SCC of the orbit is even more of a rare entity, with fewer than 15 cases being reported in the literature. To our knowledge, two of those cases have been reported after retinal detachment surgery, leading some to speculate that malignant transformation had occurred after the implantation of conjunctival epithelial cells [6,7-9]. Reports of cystic orbital SCC are even rarer than primary or secondary SCC of the orbit. To our knowledge, there are only three published cases in the literature and 10 patients that have been reported to have cystic SCC [6]. A rare variant of SCC, spindle cell carcinoma, which presents from squamous and mesenchymal cells has been reported as well in the orbit as secondary [9-11]. Only a limited number of cases have been reported to be non-apical primary orbital SCC [1-4,7]. It is critical to understand how the histopathology of the orbit contributes to the rarity of this condition. The presence of squamous epithelial cells is not native to the orbital region, thus making primary SCC a more isolated and infrequent finding [4]. The classical features are chemosis, orbital proptosis, diplopia, limitations in extraocular eye movement, and headaches [12-14]. We present a case of primary orbital SCC, an uncommon condition, detailing its diagnostic approach and treatment regimen.

## Case presentation

A 74-year-old male patient with a history of stage IV chronic obstructive pulmonary disease (COPD) and asbestosis exposure presented to the hospital due to several weeks of right eye swelling with worsening pain, headaches, and diplopia. The patient had complaints of a prominent mass visible on the superolateral side of his right orbit. Initial CT head was concerning for an infiltrating mass versus orbital cellulitis. The patient had cataract extraction with an implant of intraocular lens on both eyes approximately eight months prior. After the surgery, he noted right-sided numbness and swelling that did not subside. He started noticing blurry vision albeit using new glasses and a headache that worsened in the last month.

**Figure 1:**
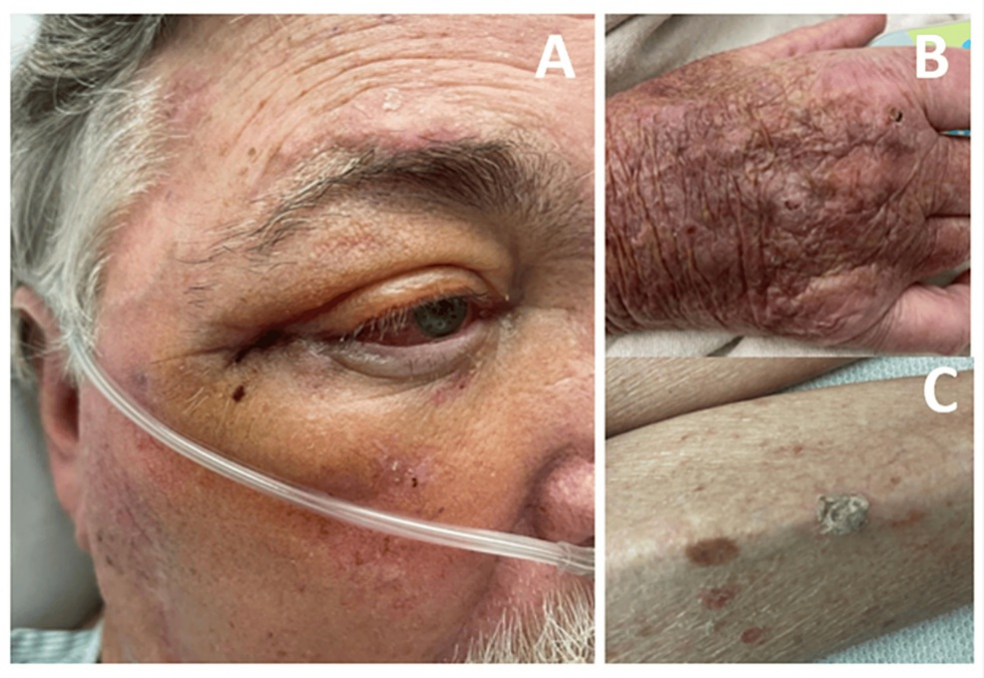
(A) Left image with a palpable mass on the superolateral side of the orbit. (B) Keratotic papule on the dorsal left hand. (C) Hyperkeratotic lesion on the shin of the right lower extremity.

On physical examination, a round, firm, fixed, and tender mass was palpable in the superolateral side of the right orbit (Figure 1A). There was moderate chemosis present. The patient demonstrated no extraocular muscle deficits in his left eye, with a distinct inability to abduct his right eye and marked diplopia. In addition, in his left dorsal hand, he had a keratotic papule (Figure 1B), the right lower extremity shin had a hyperkeratotic crusted papule (Figure 1C), and the left shoulder had a scaly pink plaque with telangiectasias.

Neurosurgery and Ophthalmology were consulted on the case to further provide guidance on management and diagnosis. The patient was started on dexamethasone to alleviate symptom burden, which resulted in the improvement of his headaches. Ophthalmology recommended obtaining magnetic resonance imaging (MRI) with contrast prior to undergoing a biopsy of the lesion.

**Figure 2:**
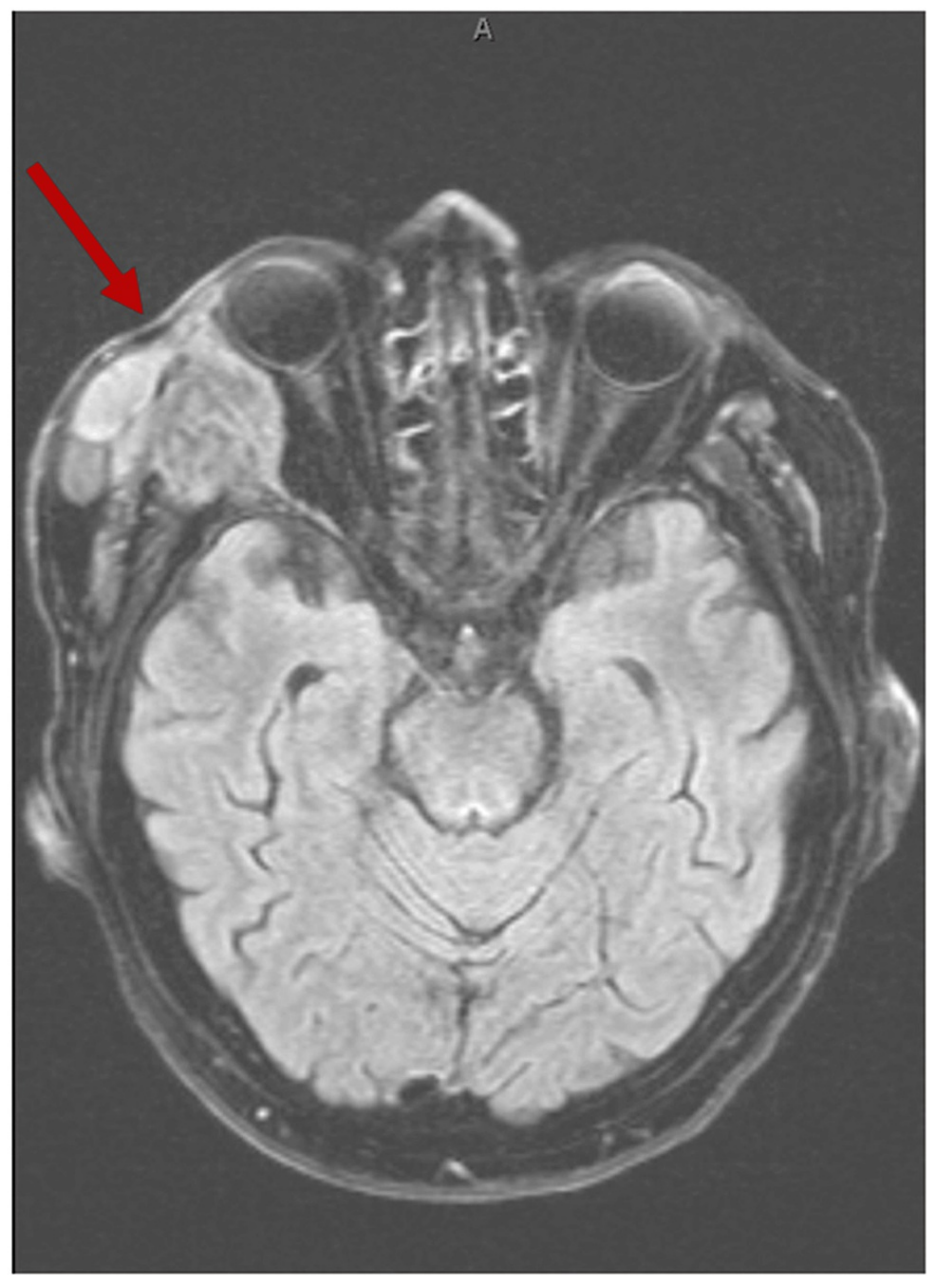
Magnetic resonance imaging (MRI) of the brain, face, orbits, and neck showing lesions in the right orbital region (shown in the arrow).

MRI with contrast done of the brain, face, orbits, and neck showed a 3.5 cm permeative lesion centered over the right sphenoid bone with underlying osseous erosion and invasion of the right lateral extraconal orbit with a medial deviation of the right lateral rectus muscle and right globe proptosis with funneling of the posterior globe (Figure 2). In addition, it reported involvement of the right temporal subcutaneous soft tissues and right temporalis musculature and intracranial extensions of the right sphenoid wing breakthrough along the anterior aspect of the right middle cranial fossa. 

**Figure 3:**
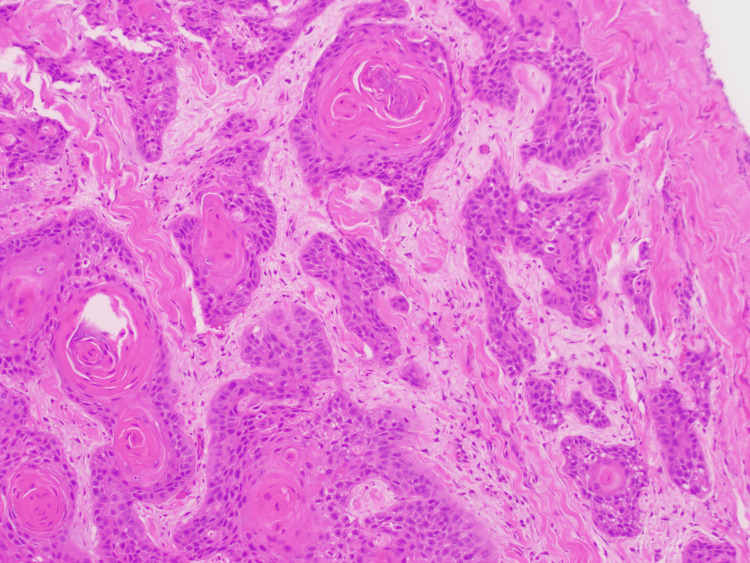
Hematoxylin and eosin (H&E), 10x, variably sized nests of squamous cells, some containing keratin pearls, which is characteristic of this neoplasm.

The patient underwent orbitotomy and biopsy by oculoplastic. During the biopsy, a keratin-like material was removed. Pathological results from the biopsy revealed moderately differentiated SCC (Figure 3). Ophthalmology recommended starting chemotherapy with irradiation and close follow-up in the clinic for surgical intervention in nine months. Oncology determined no need for emergent chemotherapy inpatient. The patient was discharged, and his management was to be guided by a multidisciplinary team consisting of oncology, radiation oncology, and ophthalmology. Immunohistochemistry (IHC) shows positivity for cytokeratin.

## Discussion

Based on a thorough review of the literature, there are less than 15 cases of primary orbital SCC reported [1-4,7]. Of those, two were atypical, and they occurred after retinal surgery [4]. The conjunctival epithelium was transplanted in those two cases, and it is presumed that there was malignant transformation of the conjunctival cyst. Several other cases report malignant transformation of a lacrimal gland or orbital cyst [3]. With respect to the location, there are only three reported cases of non-apical orbital SCC in the literature [3].

Cases of cystic orbital SCC provide additional ambiguity surrounding whether they are true cysts with fluid secretion by specific cells or if they are pseudocysts created by lymphatic blockage [6]. The presence of squamous epithelium in this region would further attest to this being a true cyst.

There have been several cases of desmoplastic SCC in the periorbital region. Desmoplasia, a host immune response to tumor cells, is an independent risk factor for metastasis and recurrence of malignancy and is an uncommon and aggressive subtype [8].

Spindle cell carcinoma, also known as carcinoma-sarcoma or sarcomatoid carcinoma, is another subtype of SCC. It is unclear whether this variant of SCC is a more aggressive form, or if the aggressive course of the tumor will depend on the origin and site of malignancy. Most spindle SCC arises from the conjunctiva [13-14]. There is a large population of pluripotent stem cells in the orbital region, and it is hypothesized that the primary spindle SCC of the orbit is a culmination of malignant degeneration of the stem cells into mesenchymal and epithelial cells [9].

Primary SCC is a diagnosis of exclusion [3]. Delays in diagnosis are typical due to the nonspecific nature of symptom presentation; headaches, swelling, localized pain, and diplopia can occur in many other more commonly occurring disorders. This results in delays in diagnosis and treatment. In this case report, the patient did have several prior cutaneous lesions, including SCC of the lower leg and hand, both of which had been excised. It is unlikely that there is an association between his previous diagnosis of SCC and his orbital SCC, as the locations are distant from each other. We can assume, albeit with moderate uncertainty, that it is unlikely that the orbital mass was a result of perineural invasion or metastasis. Thus, we can conclude that the orbital SCC is primary in nature. The lack of a surrounding keratin material in nearby structures is also supportive of our conclusion. Although there was only a temporal association, we wonder if cataract surgery could play any role in the current problem. The limitations of this report include ambiguity surrounding the true nature of his SCC and whether it is of primary or metastatic origin.

Considering that orbital squamous cell cancer is uncommon, there is not a well-recognized therapy for it. This provides additional challenges in providing an ideal course of management for the patient. In some documented cases, the treatment course included chemotherapy and radiation after surgically removing the eye and orbital radiation. In this instance, we chose to use a tissue sample for diagnosis. A multidisciplinary strategy was employed to direct therapy and management when the diagnosis was established. It was decided to have radiation and chemotherapy, with surgery to follow in nine months. Although surgical excision of the lesion with adjuvant chemotherapy and radiation is the most used approach (reported in more or less eight patients), chemotherapy followed by radiation has been attempted in two patients. There has been only one report of complete response to radiotherapy (RT) alone. The goals of RT attempted to reduce the size of the SCC, thereby reducing symptom burden. Success in using RT alone has only been reported once in the literature [7,10-12]. There are few cases reported of primary orbital SCC, further complicating the management of the patient, and how to optimize the course of treatment. Radiation, chemotherapy, and surgical excision of the lesion are previously explored treatment options. Surgical excision with and without exenterations paired with adjuvant chemotherapy has been performed as well, and they have rendered positive outcomes in their patients six months after diagnosis [4]. Orbital exenteration, craniofacial resection, tumor debulking, and chemoradiotherapy can all be attempted as well.

## Conclusions

Primary orbital SCC is a very uncommon malignancy with a clinical presentation that may provide a challenge for providing a timely diagnosis, management, and treatment. Our case demonstrated that there are many unique obstacles to navigate when facing a highly uncommon condition, as there are no gold standards for management. We hope to bring awareness to this rare condition and provide insights into different cases and presentations of this unique malignancy.
